# Standardization of the Korean Version of the Geriatric Depression Scale: Reliability, Validity, and Factor Structure

**DOI:** 10.4306/pi.2008.5.4.232

**Published:** 2008-12-31

**Authors:** Ji Yang Kim, Joon Hyuk Park, Jung Jae Lee, Yoonseok Huh, Seok Bum Lee, Seung Kyoung Han, Sung Won Choi, Dong Young Lee, Ki Woong Kim, Jong Inn Woo

**Affiliations:** 1 Department of Brain Injury Rehabilitation, National Rehabilitation Center, Seongnam, Korea.; 2 Department of Neuropsychiatry, Seoul National University Bundang Hospital, Seongnam, Korea.; 3 Jisang Psychiatric Clinic, Seongnam, Korea.; 4 Department of Psychiatry, Dankook University Hospital, Seoul, Korea.; 5 Department of Neuropsychiatry, Seoul National University Hospital, Seoul, Korea.; 6 Department of Psychiatry, Seoul National University College of Medicine, Seoul, Korea.

**Keywords:** Revised Korean version of Geriatric Depression Scale, Validity, Reliability, Factor analysis, Major depressive disorder, Minor depressive disorder

## Abstract

**Objective:**

We developed a Revised Korean version of the Geriatric Depression Scale (GDS-KR) and examined its reliability, validity, and factor structures. We also estimated its optimal cutoff scores for major depressive disorder (MDD) and minor depressive disorder (MnDD) stratified by age and education.

**Methods:**

The GDS-KR was administered to 888 subjects (61 MDD patients, 45 MnDD patients, and 782 normal elders). Its internal consistency and test-retest reliability were examined. Its concurrent validity was evaluated using Pearson correlation coefficients with the Korean version of the Center for Epidemiologic Studies Depression Scale (CES-D-K) and Hamilton Depression Rating Scale (HAM-D). The mean GDS-KR scores of the MDD patients, MnDD patients and normal elders were compared to evaluate its discriminant validity. To evaluate its construct validity, a principal component analysis with varimax rotation was performed. Receiver operator characteristic (ROC) curve analyses were performed to evaluate its diagnostic ability.

**Results:**

Chronbach's coefficient alpha for the GDS-KR was 0.90 and the test-retest reliability was 0.91 (p<0.01). The Pearson correlation coefficients of the GDS-KR scores with the CES-D-K and HAM-D scores were 0.63 (p<0.01) and 0.56 (p<0.01), respectively. The GDS-KR consisted of 5 factors. The optimal cut-off scores of the GDS-KR were 16/17 for MDD only and 15/16 for both MDD and MnDD. The optimal cutoff scores of the GDS-KR were higher in the less educated and younger subjects. The diagnostic accuracy for MDD of the GDS-KR was higher than that of the CES-D.

**Conclusion:**

The GDS-KR was found to be a reliable and valid questionnaire for screening MDD and MnDD in late life.

## Introduction

Late life depression (LLD) is one of the most prevalent psychiatric disorders in elders^1^ and is associated with increased morbidity, mortality, medical illness, and dementia. Depression is the leading cause of disability measured by Years Lived with Disability and the 4^th^ leading contributor to the global burden of disease estimated by Disability Adjusted Life Years.^2^ However, LLD is still underrecognized and undertreated due to its sub-syndromal features along with its complicated etiologies.^3^ Like major depressive disorder (MDD), subsyndromal depression (SSD) was also reported to be associated with adverse clinical outcomes, increased use of medical and mental health services, an increased risk for future pronounced mood disorders, and increased social dysfunction and disability in the elderly.^4^-^6^

The Geriatric Depression Scale (GDS)^7^ is a screening test for depression in late life that is comprised of 30 simple questions. Unlike other screening instruments for depression such as the Beck Depression Inventory (BDI),^8^ the Center for Epidemiologic Studies Depression scale (CES-D)^9^ and Zung Self-Rating Depression Scale (SDS),^10^ the GDS does not contain items regarding physical symptoms that are very prevalent in the elderly due to physical disorders. Instead, it contains questions for memory and concentration which are more common in LLD than early life depression. In addition, each item of the GDS is reported as 'Yes' or 'No', which enhances its inter-rater reliability and shortens its administration time in the elderly.

The GDS has recently been translated and used in 17 countries including Korea. Although 4 studies have been conducted for the purpose of developing the Korean version of the GDS there were several methodological limitations in these studies. First, the study subjects were inappropriately selected in some of them.^11^-^14^ For example, subjects with psychiatric disorders other than major depressive disorder were enrolled as control subjects in some studies.^12^-^14^

Second, diagnostic assessments for MDD and comorbid psychiatric conditions were limited in some studies. For example, depressive disorders were diagnosed using only Hamilton Depression Rating Scale (HAM-D) score in one previous study.^14^ Dementia, which is prevalent and frequently comorbid with LLD, was not systematically diagnosed in most of the previous studies.^11^,^12^,^14^

Third, several items were mistranslated. For example, helpless and hopeless were not clearly distinguished in the Korean version of the GDS standardized by Cho et al.^13^

Fourth, the sensitivity and specificity of the previous versions (60% to 72%)^11^,^12^,^14^ were lower than those of the other language versions,^15^ indicating that the diagnostic abilities of these previous versions were limited.

Fifth, the factor structure was different between the previous versions, which may be attributed to the differences in the study samples and translations.^12^,^13^ It was also different from that of the original version of GDS.^12^

Sixth, the GDS score can be influenced by the demographic factors of the subjects, such as their age and educational level, since it is a self-administered questionnaire. However, the demographic influences on the diagnostic ability of the GDS were not examined in previous studies.^11^-^14^ Seventh, diagnostic accuracy of the GDS for subsyndromal depression such as minor depressive disorder (MnDD) has never been studied yet despite it is common in the elderly.

Therefore, we developed a revised Korean version of the GDS (GDS-KR) and examined its reliability and validity. We also estimated the education- and age-stratified optimal cutoff scores for both MDD and MnDD.

## Methods

### Translation of the Geriatric Depression Scale

The original English version of the GDS^7^ was translated into Korean by a panel of two psychiatrists and one clinical psychologist who are familiar with both Korean and English.

Seven Korean psychiatrists who were familiar with both English and Korean reviewed the first translated version, and reworded and reformulated some items to minimize the difference from the original version. This second translated version of the GDS was preliminarily applied to 50 LLD patients (age=75.1±6.6 years old) and 50 normal elders (age=73.3±5.3 years old) from September 2001 to August 2002.

According to the results obtained from the preliminary application and analysis, several items were modified to improve the comprehensibility and applicability. This third translated version of the GDS was applied to 57 MDD patients (age=68.9±7.2 years old) and 109 normal elders (age=68.5±6.9 years old) from September 2004 to December 2004.

According to the results obtained from the second preliminary application and analysis, the fourth translated version was made by a panel of four psychiatrists and one clinical psychologist.

Then, the fourth translated version was back-translated into English by an expert translator who was not familiar with the original GDS. We discussed the original GDS version and the back-translated English version with the expert translator. Some minor modifications were made after this discussion and this resulted in the final Korean version of the GDS which was to be subjected to a validity and reliability study.

### Subjects

The subjects with LLD including both MDD and MnDD were recruited either from the visitors to the Geropsychiatry Clinic of the Seoul National University Bundang Hospital or from the community-dwelling elderly who participated in the Korean Longitudinal Study on Health and Aging (KLoSHA).^16^ Control subjects were recruited from the community-dwelling elderly included in the KLoSHA. All of the subjects were aged 50 or over.

The subjects who were diagnosed to have MDD or MnDD were enrolled in the patient group, while those that did not were enrolled in the control group. The subjects who had other major psychiatric disorders such as dementia and anxiety disorders were excluded from both the patient and control groups. The subjects who had serious medical or neurological disorders that could affect their mood and cognition were also excluded.

### Diagnosis

Standardized clinical interviews, physical and neurological examinations were administered to all subjects using the Korean version of Mini-International Neuropsychiatric Interview (MINI)^17^ and the Korean version of the Consortium to Establish a Registry of Alzheimer's Disease assessment battery (CERAD-K)^18^ by a psychiatrist with advanced training in neuropsychiatry and dementia research. Major depressive disorder, dementia, and other Axis I major psychiatric disorders were diagnosed according to the fourth edition of the Diagnostic and Statistical manual of Mental Disorders (DSM-IV) criteria,^19^ and minor depressive disorder according to the research criteria proposed in appendix B of the DSM-IV criteria.

### Reliability

In order to evaluate the test-retest reliability, the GDS-KR was administered twice to 20 subjects with MDD or MnDD by the same rater. The test-retest interval was 1-6 days. To assess the test-retest reliability of the GDS-KR, the Pearson correlation coefficients were calculated. Its internal consistency was examined by Cronbach alpha and item-total correlations.

### Validity

To evaluate the discriminant validity, the mean GDS-KR scores were compared among the control group, MnDD group, and MDD group using analysis of variance (ANOVA) after adjusting for age, gender and educational level. To evaluate the concurrent validity, the Korean version of the CES-D-K^20^ and 17-item HAM-D^21^ were administered together and the Pearson correlation coefficients with the GDSKR were calculated. An exploratory factor analysis was performed using principal component analysis with varimax rotation to determine the factor structure of the GDS-KR.

The optimal cut-off scores satisfying both the sensitivity and specificity of the GDS-KR, CES-D-K, and HAM-D for MDD or MnDD were determined by receiver operator characteristic (ROC) analyses. To measure the diagnostic accuracy of each scale for MDD and MnDD, the area under the ROC curves (AUC), standard errors (SE), and 95% confidence interval (95% C.I.) were calculated. The AUC ranges between 0.5 and 1; the nearer it is to 0.5 the less accuracy it has, whereas the nearer it is to 1, the more accuracy it has.

To examine the difference in the diagnostic accuracy of the GDS-KR, CES-D-K, and HAM-D for MDD and MnDD, we compared the AUCs by calculating the critical ratio z proposed by Hanley and McNeil.^22^ The z ratio was defined as


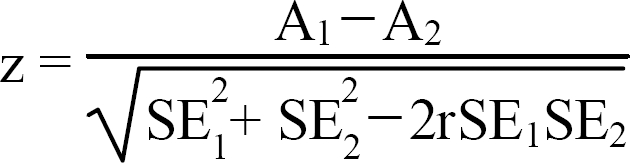


where A_1_ and SE_1_ refer to the observed AUC and estimated standard error of the AUC associated with test 1, respectively, A_2_ and SE_2_ refer to the observed AUC and estimated standard error of the AUC associated with test 2, respectively, and r refers to the estimated correlation coefficient between A_1_ and A_2_. Note that the z ratio follows the standard normal distribution.

All statistical analyses were done using SPSS (version 15.0) for Windows.

## Results

### Subjects

Finally, 888 subjects (61 MDD patients, 45 MnDD patients, 782 control subjects) were enrolled in the present study. The demographic characteristics of the subjects are summarized in Table 1.

### Reliability

The GDS-KR was found to have an excellent degree of internal consistency. Chronbach's coefficient alpha for the GDS-KR was 0.90. The item-total correlations were also significant (p<0.01, Pearson correlation tests) and high for all questions other than question 29 (Is it easy for you to make decisions?), ranging from 0.26 (Do you prefer to avoid social gatherings?) to 0.64 (Do you feel downhearted and blue?). The test-retest reliability was 0.91 (p<0.01) indicating that the performance of the GDS-KR is highly stable over time.

### Validity

The Pearson correlation coefficients of the GDS-KR with CES-D-K and HAM-D were 0.63 (p<0.01) and 0.56 (p<0.01), respectively, indicating that the GDS-KR has good concurrent validity.

The GDS-KR scores were significantly different between the three diagnostic groups (F[2, 885]=116.77, p<0.05). In the post-hoc analysis, the GDS-KR score of the MDD group was significantly higher than those of the MnDD group (F=3.51, p<0.05) and the control group (F=-11.93, p<0.001), and that of the MnDD group was higher than that of the control group (F=-8.41, p<0.001), indicating that the GDS score could validly discriminate MnDD from euthymia and MDD (Table 2).

### Factor analysis

The factor analysis yielded five factors in the GDS-KR accounting for 47.37% of the variability (Table 3). Factor 1 included 11 items ('Are you bothered by thoughts you can't get out of your head?', 'Do you worry a lot about the past?', 'Do you often get restless and fidgety?', 'Are you afraid that something bad is going to happen to you?', 'Do you frequently worry about the future?', 'Do you frequently get upset over little things?', 'Do you feel downhearted and blue?', 'Do you frequently feel like crying?', 'Do you often feel helpless?', 'Do you often get bored?', 'Do you feel that your life is empty?'), and accounted for 29.17% of the total variance. We named this factor 'sad mood and agitation'.

Factor 2 included 9 items ('Are you basically satisfied with your life?', 'Do you feel happy most of the time?', 'Are you in good spirits most of the time?', 'Do you think it is wonderful to be alive now?', 'Do you find life very exciting?', 'Do you feel that your situation is hopeless?', 'Do you enjoy getting up in the morning?', 'Are you hopeful about the future?', 'Do you think that most people are better off than you are?'), and accounted for 5.34% of the total variance. We named this factor 'positive mood'.

Factor 3 included 4 items ('Have you dropped many of your activities and interests?', 'Do you feel full of energy?', 'Is it hard for you to get started on new projects?', 'Do you feel pretty worthless the way you are now?'), and accounted for 4.97% of the total variance. We named this factor 'lack of energy'.

Factor 4 included 3 items ('Do you feel you have more problems with memory than most?', 'Do you have trouble concentrating?', 'Is your mind as clear as it used to be?'), and accounted for 4.02% of the total variance. We named this factor 'cognitive inefficiency'.

Factor 5 included 2 items ('Do you prefer to stay at home, rather than going out and doing new things?', 'Do you prefer to avoid social gatherings?'), and accounted for 3.87% of the total variance. We named this factor 'social withdrawal'.

### Diagnostic accuracy and optimal cutoff scores

As shown in Table 4, the AUCs of the GDS-KR, CES-D-K, and HAM-D were greater than 0.80, indicating that all three tests are useful for detecting LLD.

The AUC of the GDS-KR (AUC_GDS-KR_) was bigger than that of the CES-D-K (AUC_CES-D-K_), and the difference reached statistical significance (z=3.68, p<0.0001 for MDD only; z=4.00, p<0.0001, for both MDD and MnDD), indicating that the GDS-KR is more accurate than the CES-D-K in diagnosing LLD. However, the AUC of GDS-KR was lower than that of HAM-D (z=5.79, p<0.0001 for MDD only; z=6.73, p<0.0001, for both MDD and MnDD).

As shown in Table 5, AUC_GDS-KR_ was lower in the less educated subjects than in the more highly educated subjects. The optimal cut-off score of the GDS-KR was 16/17 for MDD only and 15/16 for both MDD and MnDD. The optimal cutoff scores of the GDS-KR were higher in the less educated and younger subjects.

## Discussion

This study attempted to evaluate the diagnostic value of the GDS-KR in LLD, which is one of the most important mental disorders in the elderly, by assessing its reliability and validity. The GDS-KR showed good internal consistency. It also showed significant positive correlations with other depression-screening instruments including the CES-D and the HAM-D, thus confirming its validity. In addition, we tested the differences in the GDS-KR score in terms of the educational level as a covariate between the normal control, major depression and minor depressive disorder groups. Since our result showed that there were significant differences in the GDS-KR score between these 3 groups, the GDS-KR can be used to distinguish between late-life depressive disorders, particularly major depression and minor depressive disorder.

In the present study, the cutoff score of the GDS-KR for MDD was 16/17, where the sensitivity and specificity were 81.22% and 87.18%, respectively. Although we did not directly compare the diagnostic abilities of the GDS-KR and previous Korean versions of GDS, the former may have better diagnostic ability than the latter, since the sensitivities and specificities of the previous Korean versions were 60-72%.^11^-^14^ The cutoff score of the GDS-KR for LLD including both MDD and MnDD was 1 point lower than that for MDD only (15/16), where the sensitivity and specificity were 82.0% and 78.11%, respectively. This indicated that the GDS-KR may be a good screening test not only for MDD but also for MnDD in late life.

As far as we know, this is the first study that showed the influence of age and education on the cutoff score of the GDS for MDD and the estimated age- and education-stratified cutoff scores for LLD. Demographic factors such as education and age may influence the score of self-administered questionnaires such as the GDS. However, the cutoff scores for MDD stratified by demographic factors have never been studied yet. The cutoff scores of the GDS-KR for late life depression were 1-2 points higher in the younger and less educated elders in the present study. The diagnostic accuracy for LLD did not significantly differ by age, but was lower in the less educated elders. These age- and education-stratified cutoff points of the GDS-KR may contribute to enhance the accuracy of screening late life depression in both clinical and research settings in the future.

In addition, the diagnostic accuracy for MDD of the GDS-KR was significantly higher than that of the CES-D, indicating that the former may be better in screening MDD in late life than the latter. Considering that the GDS's yes or no format may ease administration, the GDS seems to be a better instrument for screening MDD than the CES-D, at least in late life.

The GDS-KR was meaningfully factored into five clearly separated factors (sad mood and agitation, positive mood, lack of energy, cognitive inefficiency, social withdrawal). This factor structure is quite similar to that of the original English version of GDS^23^ except that the 'cognitive inefficiency' factor was extracted as a separate factor in the GDS-KR. The original English version of GDS was reported to be comprised of five factors including sad mood (8, 6, 23, 13, 16, 18, 10, 24, 22), lack of energy (29, 20, 21, 30, 25, 2), positive mood (15, 27, 9, 5, 7, 19), agitation (24, 11, 4), and social withdrawal (12, 28).^7^ The factor structures of the previous Korean versions of GDS were somewhat different from those of the GDS-KR and the original English version of GDS. Three factors (anti-vitality, depression, cognitive function) explained 43.1% of the total variance in the version of Kee et al.,^11^ and 7 factors (core depressive feature, loss of interest/pleasure, feeling of unhappiness, agitation, cognitive inefficiency, social withdrawal tendency, lack of motivation) explained 53.4% of the total variance in Jung et al.^12^ However, in contrast to the original English version of GDS, the cognitive inefficiency was extracted as a separate factor in all of the Korean versions of GDS. The items of the Korean versions of GDS loaded in the cognitive inefficiency factor were loaded in the 'lack of energy' factor in the original English version. These differences in the factor structure of GDS between Koreans and Caucasians may be attributed to cultural differences in depressive mood and cognition in the elderly and sociodemographic differences between the study samples. In the present study, item 29 ("Is it easy for you to make decisions?") was not loaded in any of the five factors. This item was categorized as 'lack of energy' in the version of Kee et al.^11^ and in the original English version,^23^ 'agitation' in the version of Jung et al.,^12^ and 'concern and anxiety' in the version of Rhyoo et al.^25^ Considering that depressive disorders and anxiety disorders have indecisiveness as their common symptom, item 29 can reflect the cognitive inefficiency associated with either depression or anxiety of the subject. Further studies are required to confirm whether item 29 can serve as an appropriate item for screening depression.

The major strengths of this study are as follows. First, all of the participants were assessed directly by expert neuropsychiatrists, conformed to standardized and structured instruments for diagnosing dementia, stroke, and major psychiatric disorders. These practices may have enhanced the diagnostic accuracy of comorbid neuropsychiatric conditions. This thorough exclusion of comorbid neuropsychiatric disorders might have contributed to enhance the diagnostic accuracy indices of the GDS-KR. Second, the cutoff scores for LLD were estimated based on the severity of depression (MDD, MnDD), age, and education, which may enhance the usefulness of the GDS-KR in both clinical and research settings. One major limitation of the present study was the sample size. The sample size was not large enough to examine the influences of the various sociodemographic variables other than age and education.

In conclusion, the GDS-KR was found to be a valid and reliable screening instrument for MDD and MnDD in Korean elders, regardless of their age and educational level.

## Figures and Tables

**TABLE 1:**
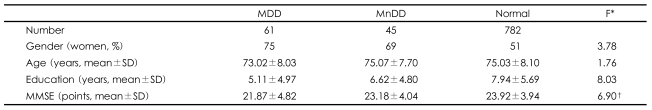
Demographic characteristics of the subjects

^*^χ^2^ for categorical variables, F for continuous variables, ^†^p<0.01. MDD: major depressive disorder, MnDD: minor depressive disorder, MMSE: mini mental status exam, SD: standard deviation

**TABLE 2:**
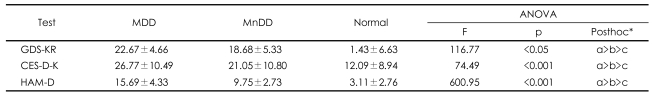
The scores of the Revised Korean version of the Geriatric Depression Scale (GDS-KR), the Korean version of the Center for Epidemiologic Studies Depression Scale (CES-D-K), and the Hamilton Depression Rating Scale (HAM-D) scores stratified by diagnoses

^*^Scheffe posthoc comparison. a: MDD, b: MnDD, c: normal. MDD: major depressive disorder, MnDD: minor depressive disorder, ANOVA: analysis of variance

**TABLE 3:**
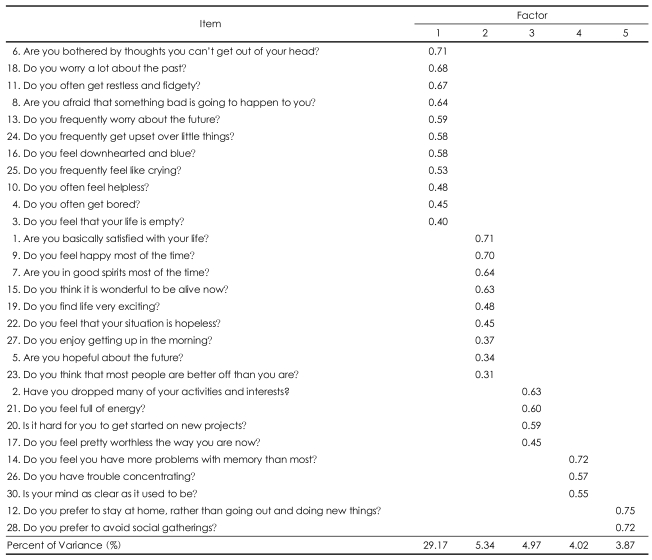
Factor structure of the Revised Korean version of the Geriatric Depression Scale (GDS-KR)

**TABLE 4:**
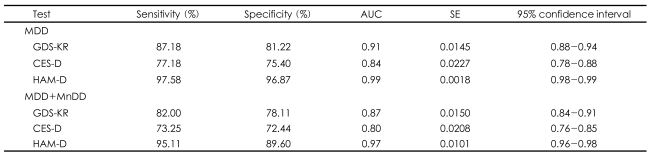
Diagnostic accuracy indices of the Revised Korean version of the Geriatric Depression Scale (GDS-KR) for major depressive disorder (MDD) and minor depressive disorder (MnDD)

CES-D: Center for Epidemiologic Studies Depression scale, HAM-D: Hamilton Depression Rating scale

**TABLE 5:**
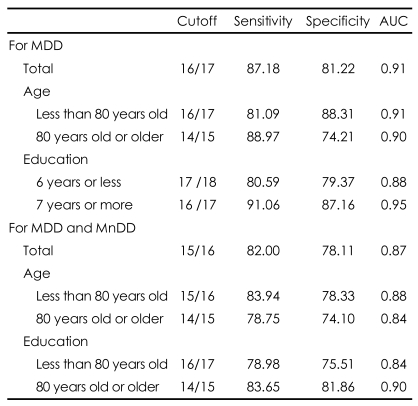
The values of the accuracy indices according to various cut-off scores for the Revised Korean version of the Geriatric Depression Scale (GDS-KR) of major depressive disorder (MDD) and minor depressive disorder (MnDD)

AUC: under the ROC curves, ROC: receiver operator characteristic
